# Performance of large language models ChatGPT and Gemini in child and adolescent psychiatry knowledge assessment

**DOI:** 10.1371/journal.pone.0332917

**Published:** 2025-09-19

**Authors:** Johanna Charlotte Neubauer, Anna Kaiser, Leon Lettermann, Tobias Volkert, Alexander Häge

**Affiliations:** 1 Central Institute of Mental Health, Mannheim, Baden-Württemberg, Germany; 2 Department of Child and Adolescent Psychiatry/Psychotherapy, University Hospital of Ulm, University of Ulm, Ulm, Germany; 3 Bioquant-Center & Institute for Theoretical Physics, Heidelberg University, Heidelberg, Baden-Württemberg, Germany; 4 Independent Researcher, Heidelberg, Baden-Württemberg, Germany; South China Normal University, CHINA

## Abstract

**Objective:**

This study evaluates the performance of four large language models—ChatGPT 4o, ChatGPT o1-mini, Gemini 2.0 Flash, and Gemini 1.5 Flash—in answering multiple-choice questions in child and adolescent psychiatry to assess their level of factual knowledge in the field.

**Methods:**

A total of 150 standardized multiple-choice questions from a specialty board review study guide were selected, ensuring a representative distribution across different topics. Each question had five possible answers, with only one correct option. To account for the stochastic nature of large language models, each question was asked 10 times with randomized answer orders to minimize known biases. Accuracy for each question was assessed as the percentage of correct answers across 10 requests. We calculated the mean accuracy for each model and performed statistical comparisons using paired t-tests to evaluate differences between Gemini 2.0 Flash and Gemini 1.5 Flash, as well as between Gemini 2.0 Flash and both ChatGPT 4o and ChatGPT o1-mini. As a post-hoc exploration, we identified questions with an accuracy below 10% across all models to highlight areas of particularly low performance.

**Results:**

The accuracy of the tested models ranged from 68.3% to 78.9%. Both ChatGPT and Gemini demonstrated generally solid performance in the assessment of in child and adolescent psychiatry knowledge, with variations between models and topics. The superior performance of Gemini 2.0 Flash compared with its predecessor, Gemini 1.5 Flash, may reflect advancements in artificial intelligence capabilities. Certain topics, such as psychopharmacology, posed greater challenges compared to disorders with well-defined diagnostic criteria, such as schizophrenia or eating disorders.

**Conclusion:**

While the results indicate that language models can support knowledge acquisition in child and adolescent psychiatry, limitations remain. Variability in accuracy across different topics, potential biases, and risks of misinterpretation must be carefully considered before implementing these models in clinical decision-making.

## Introduction

Large language models (LLMs) such as ChatGPT or Gemini are artificial intelligence (AI) systems developed to understand and generate human-like text. While LLMs hold significant potential to transform medical science and practice, their widespread use raises critical questions about accuracy, bias, and data privacy. In certain fields of medicine, AI has already demonstrated remarkable diagnostic accuracy, for example in the detection of melanomas [1]. In other areas, such as psychiatry and psychology, AI is increasingly used in therapeutic contexts, including digital health applications [2] for several targets such as (psycho-)education, monitoring and mental health diagnostics [3]. The integration of LLMs into psychiatry and psychology/psychotherapy offers promising options: improving shared decision-making and therapy, expanding access to care, enhancing patient autonomy, and potentially reducing health disparities [4]. However, concerns about inaccurate outputs (for instance concerning therapy plans or pharmacotherapy), limited user comprehension, misinterpretations and ethical implications are crucial [4].

Multiple choice questions (MCQs) are a useful tool for assessing and comparing individual performance across a broad range of topics in an objective and standardized manner. In this study, we aimed to evaluate the performance of four LLMs in answering MCQs in the field of child and adolescent psychiatry – questions originally designed to assess human knowledge. While LLM performance on MCQs has already been documented in medical fields such as dermatology, ophthalmology or internal medicine [5–7], to our knowledge no data exists on their performance in child and adolescent psychiatry.

To address this gap, we tested two versions of ChatGPT and two versions of Gemini using 150 MCQs from a study guide for the specialty board review in child and adolescent psychiatry.

We selected ChatGPT 4o and Gemini 2.0 Flash as widely used default models offered by OpenAI and Google AI respectively. For comparison to an affordable reasoning model we selected ChatGPT o1-mini, and to compare development over one generation we compared Gemini 2.0 Flash with Gemini 1.5 Flash. The details of the models used are summarized in the S2 Table.

ChatGPT 4o is the current default ChatGPT model, versatile and fast for many different use cases and questions.

Gemini 2.0 Flash is a more advanced version of Gemini 1.5 Flash, offering improvements in speed, performance and multimodal capabilities.

GPT o1-mini is a smaller and faster version of the more complex o1 model, developed for complex reasoning tasks, coding and problem solving. It stands for a high performance in creative and analytical tasks, making it reasonable for research topics and up-to-date information.

Gemini 1.5 Flash represents fast response times and good general knowledge, optimized for real-time AI applications.

Our primary research objective was to compare the accuracy of ChatGPT 4o, ChatGPT o1-mini, Gemini 2.0 Flash and Gemini 1.5 Flash in answering MCQs relevant to the child and adolescent psychiatry board review. Moreover, we conducted a post-hoc exploration to examine performance across different subtopics within the field.

## Methods

We used 150 standardized MCQs from the fifth edition of the study guide “Child and Adolescent Psychiatry for the Specialty Board Review” by Caitlin R. Costello and Lauren T. Schumacher [8]. These questions are designed to help candidates prepare for the specialty board examination in child and adolescent psychiatry. A sample of questions was carefully selected from each chapter of the book by expert validation. Questions related to the organization or structure of the U.S. medical healthcare system, U.S.-specific epidemiological data and questions regarding the U.S. system of justice were excluded. Moreover, we excluded questions involving diagnostic or therapeutic concepts not commonly used in our clinical setting, as well as questions focusing on FDA-approved treatments.

Each question consisted of five different answers with only one correct option. The distribution of questions across chapters is detailed in Table 1. We submitted the questions to ChatGPT and Gemini between 2025/03/11–2025/03/13. Only text based questions were used. Ethics approval was not required for this study, as we only used publicly available data without involving human participants.

**Table 1:** Distribution into chapters of total 150 MCQs.

	Distribution into chapters	Numbers of MCQs
1	Normal Growth and Development	19
2	Neurodevelopmental Disorders	15
3	Schizophrenia and Other Psychotic Disorders	6
4	Bipolar and Related Disorders and Depressive Disorders	9
5	Disruptive, Impulse-Control, and Conduct Disorders	5
6	Anxiety Disorders, Obsessive-Compulsive and Related Disorders,Trauma- and Stressor-Related Disorders, and Dissociative Disorders	9
7	Feeding and Eating Disorders, Elimination Disorders, and Obesity	11
8	Somatic Symptoms and Related Disorders and Sleep–Wake Disorders	5
9	Substance-Related and Addictive Disorders	6
10	Special Issues (Diverse Populations, Medically Ill Children, Suicide, and Abuse)	10
11	Psychological Testing and Rating Scales	3
12	Psychopharmacology and Medication-Induced Movement Disorders and Other Adverse Effects of Medication	14
13	Psychotherapies	7
14	Treatment Settings	4
15	Special Topics (Consultation, Forensics, and Public Health)	3
16	Research Design, Statistics, and Technologies	7
	Case reports	17

We used ChatGPT 4o, ChatGPT o1-mini, Gemini 2.0 Flash and Gemini 1.5 Flash. To account for the stochastic nature of LLMs, we presented each question ten times, randomizing the order of the answer choices in each instance. This approach aimed to minimize known biases that LLMs may exhibit when responding to MCQs [9]. For each question, accuracy was defined as the proportion of correct answers out of ten attempts, resulting in a score ranging from 0% to 100%. We calculated the mean value of the accuracies of each LLM and compared the performance of the two versions of Gemini and of Gemini 2.0 Flash with ChatGPT o1-mini and of Gemini 2.0 Flash with ChatGPT 4o respectively. For this purpose we performed statistical analysis on SPSS using paired t-tests. In a post-hoc exploration, we identified questions with an accuracy below 10% in all LLMs, aiming to highlight specific content areas with particular low model performances.

## Results

### Mean of accuracies in different LLMs

**Fig 1:**
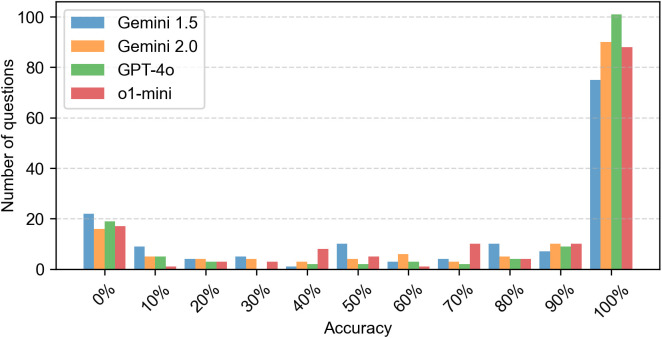
Number of questions with the corresponding detected accuracy. Gemini 1.5 Flash revealed a mean accuracy of 68.3% (SD = 39.7), Gemini 2.0 Flash 76.3% (SD = 36.4), ChatGPT o1-mini 76.7% (SD = 35.3) and ChatGPT 4o 78.9% (SD = 37.0).

Fig 1 shows the distribution of accuracy (in percent) for the four tested LLMs. All models demonstrated a similar spread of accuracy scores ranging from 0% to 100%. Descriptively, each model showed a comparable number of questions with 0% accuracy, while the majority of questions were answered correctly in all attempts by all models, resulting in 100% accuracy.

### Gemini 2.0 Flash shows better performance than Gemini 1.5 Flash

Comparing the accuracy of the models via t-tests revealed no significant differences except for the comparison between Gemini 1.5 Flash and Gemini 2.0 Flash (t = −3.004, p = .003, d = −0.245). Although the Shapiro–Wilk test indicated a significant deviation from normality, the large sample size (n = 150) and the Central Limit Theorem support the robustness of the paired t-test in this case. Nevertheless, the non-parametric Wilcoxon signed-rank test was also performed, with both approaches yielding consistent results. Normality assessments (histograms, boxplots) and Wilcoxon results are provided in the Supporting Information. Significant correlations were identified between the performance of all tested models (Gemini 1.5 Flash and Gemini 2.0 Flash: r = .641, p < .001; ChatGPT o1-mini and ChatGPT 4o: r = .667, p < .001; ChatGPT o1-mini and Gemini 2.0 Flash: r = .708, p < .001; ChatGPT 4o and Gemini 2.0 Flash: r = .756, p < .001).

### Post-hoc exploration: Similar accuracies across different chapters among tested LLMs

**Fig 2:**
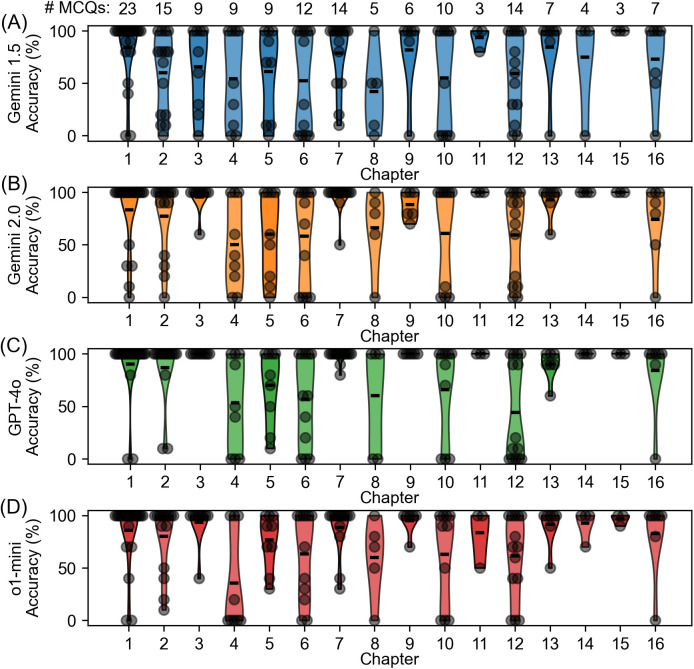
Accuracy of the tested LLMs divided into different chapters. Each grey dot illustrates the accuracy for one asked question. An accuracy of 0 represents 0/10 correct answers for one question. The headings of the chapters corresponding to their number can be found in Table 1. (A) represents the accuracy by chapter for Gemini 1.5 Flash, (B) for Gemini 2.0 Flash, (C) for ChatGPT 4o, (D) for ChatGPT o1-mini.

Fig 2 illustrates the accuracy for the different chapters. Descriptively, we see a similar distribution of accuracies in the LLMs Gemini 2.0 Flash, ChatGPT o1-mini and ChatGPT 4o, differing from Gemini 1.5 Flash. Regarding chapters 3, 7, 9, 11, 13, 14 and 15 we identified a relatively homogeneous distribution of a high accuracy across the three LLMs Gemini 2.0 Flash, ChatGPT o1-mini and ChatGPT 4o. When calculating the mean accuracy for each question across the four LLMs, and found a mean accuracy below 10% in 12 questions. These questions are listed in the S1 Table.

## Discussion

The performance of the four tested LLMs in answering MCQs in child and adolescent psychiatry ranged from 68.3% to 78.9% of correctly answered questions. The results demonstrate minor variations between the models and across different topics. Notably, Gemini 2.0 Flash outperforms the earlier version Gemini 1.5 Flash, which may reflect advancements in artificial intelligence capabilities. Furthermore, topics like psychopharmacology appear to be more challenging than questions on specific disorders such as schizophrenia or eating disorders. Still, there are limiting factors of this study which need to be discussed.

Language models such as ChatGPT and Gemini are increasingly capturing the interest of students, clinicians, researchers and the general public across various domains including the medical area. To evaluate the performance of these LLMs in child and adolescent psychiatry, we used MCQs. MCQs are a wildly used and effective method for assessing and comparing individuals in training. Therefore, the majority of medical exams consist of MCQ.

However, there is ongoing debate regarding the use of MCQs in medicine, and in particular in psychiatry, where patient evaluation through clinical exploration is essential. Unlike MCQs, which rely on predefined answers, clinical evaluation requires nuanced judgement that cannot always be encapsulated by a single correct option. Assessing mental health conditions often requires recognizing subtle variations in symptoms, which MCQs may exaggerate or oversimplify. Furthermore, in psychiatry, symptom overlap between diagnoses (e.g., depression, anxiety, personality disorders) complicates the creating of definitive answers. Moreover, proficiency in MCQ-based testing does not necessarily reflect clinical competence, as such formats fail to capture essential skills for psychiatric practice, including building a therapeutic alliance, interpreting patient narratives, and adapting diagnostic reasoning to the unique psychosocial context of each case.

Nevertheless, MCQs offer several advantages for knowledge assessment. They provide an objective scoring method, which eliminates subjectivity, and they can cover a wide range of topics within the relevant field. This makes MCQs an efficient method for testing knowledge, as they allow for the evaluation of numerous questions and the provision of rapid results. As the sorting in wrong and correct answers is not subjective, MCQs offer an advantage over methods like written texts or oral exams. Furthermore MCQs can span from basis to complex content, making them valuable for assessing a broad spectrum of knowledge.

To our knowledge, this is the first study to evaluate the performance of ChatGPT and Gemini on MCQ in child and adolescent psychiatry. Comparable studies evaluating performance accuracy in other fields, such as internal medicine or ophthalmology, have been published [5,6]. In ophthalmology, Mihalache et al. (2023) challenged ChatGPT (version not specified) with 125 MCQs from a board certification exam preparation, where the model answered 46% of the questions correctly [6]. In internal medicine, Meo et al. used 100 MCQs from endocrinology, diabetes, and diabetes technology to test both ChatGPT (version not specified) and Google’s Bard (an earlier version of Gemini). They found that ChatGPT correctly answered 52% of the questions, while Google’s Bard achieved 49% correct answers [5].

Compared with results in internal medicine and ophthalmology, our findings suggest that LLMs may achieve relatively higher performance on MCQs in child and adolescent psychiatry, possibly reflecting ongoing advancements in AI. Mean accuracies were 68.3% for Gemini 1.5 Flash, 76.3% for Gemini 2.0 Flash, 76.7% for ChatGPT o1-mini, and 78.9% for ChatGPT 4o, indicating generally solid performance. Notably, these models performed well on assessments intended for clinicians with several years of training. When contextualized with human data, a study of child and adolescent psychiatry fellows from 2007 to 2012 reported mean exam scores of 77–78% correct and a first-attempt pass rate of 96%, suggesting that the performance of Gemini 2.0 Flash, ChatGPT o1-mini, and ChatGPT 4o may be roughly comparable to that of human examinees from this period [10]. Sensitivity analyses showed significant correlations in accuracy across models, indicating broadly similar performance levels. Gemini 2.0 Flash outperformed its predecessor, Gemini 1.5 Flash, pointing toward potential improvements in this model version, though caution is warranted in interpreting these comparisons.

We investigated performance accuracy across different chapters, revealing notable differences in the ability to select correct answers depending on the chapter content. The distribution across the chapters of accuracies was quite similar in the three models Gemini 2.0 Flash, ChatGPT o1-mini and ChatGPT 4o (Fig 2). We identified chapters with higher accuracies such as chapter 3 (“Schizophrenia and Other Psychotic Disorders”), chapter 7 (“Feeding and Eating Disorders, Elimination Disorders, and Obesity“), chapter 9 (“Substance-Related and Addictive Disorders”), chapter 11 (“Psychological Testing and Rating Scales”) chapter 13 (“Psychotherapies”), 14 (“Treatment Settings”) and 15 (“Special Topics (Consultation, Forensics, and Public Health”), whereas a lower performance was found for chapter 4 (“Bipolar and Related Disorders and Depressive Disorders”), chapter 6 (“Anxiety Disorders, Obsessive-Compulsive and Related Disorders,Trauma- and Stressor-Related Disorders, and Dissociative Disorders”) and chapter 12 (“Psychopharmacology and Medication-Induced Movement Disorders and Other Adverse Effects of Medication”).

Upon examining chapters with higher accuracies, it appears that diagnostic criteria in these topics are clearer and easier to define (psychotic disorders, eating disorders) compared to conditions like disruptive disorders, depressive or anxiety disorders, where symptom overlap is more common. The high performance in the chapters on psychotherapies and treatment settings may be due to the fact that these questions primarily target surface-level knowledge rather than addressing the complexities of therapeutic practice (for example “Which of the following specific intervention(s) is (are) commonly used in CBT for depression?”) [8]. Capturing such depth in a multiple-choice format can be challenging. Therefore, this finding should not be interpreted as support for the notion that LLMs are capable of planning psychotherapies or implementing treatment plans.

In a post-hoc-exploration we identified chapters where all four tested LLMs demonstrated poor performance. These included questions on child and adolescent neurodevelopment, psychopharmacology, and assessment and diagnostic evaluation. Notably, LLMs frequently select the same incorrect answer across repeated inputs, which, in some cases, may reflect inconsistencies within the underlying literature. Additionally, we identified one incorrectly answered question in the source material itself. This highlights a known limitation of MCQs, where flawed questions are often only recognized after the exam and must then be excluded from scoring.

We were unable to determine specific reasons for the poor performance regarding psychopharmacology. However, the current state of research in pediatric psychopharmacology is complex, limited and heterogeneous, which could reflect the models´ difficulties in finding the correct answer. The poor performance in assessment and diagnostic evaluation may be explained by the fact, that assessments in children and adolescents require a deep understanding of developmental stages accompanied by clinical intuition and expertise – abilities that LLMs inherently lack. However, the small number of questions per subcategory limits the ability to draw conclusions.

Recent research highlights that LLMs can exhibit cultural biases, influenced by interaction language, user background, and the model’s own assumptions [11,12]. These biases may affect responses, underscoring the need for caution when interpreting LLM-generated results.

Moreover, ethical use of LLMs in child and adolescent psychiatry requires careful consideration of risks, including hallucinated references and clinically unsafe recommendations that could harm vulnerable patients. AI-generated content should always be reviewed by qualified professionals, with safeguards such as human oversight, accuracy checks, and transparent disclosure to ensure safe and responsible integration into clinical practice.

Further studies should include a larger number of questions, to increase the reliability of the conclusions on specific topics, incorporating open-ended questions.

In conclusion, our results suggest that both Gemini and ChatGPT performed solidly overall in the child and adolescent psychiatry knowledge assessment. Our findings indicate better performance accuracy for more advanced models such as Gemini 2.0 Flash, and highlight areas where LLMs perform less effectively. These results underscore the need for caution when using LLMs, particularly in areas like diagnostics and treatment planning in child and adolescent psychiatry. Further studies should evaluate LLMs using not only multiple-choice questions but also open-ended and case-based assessments, in order to provide a more complete understanding of their capabilities in this field. Future research could investigate how LLMs perform with complex clinical cases that require nuanced differential diagnoses, rather than relying solely on MCQs. While LLMs achieved 68.3–78.9% accuracy on MCQs, this study did not directly benchmark model performance against human reference scores (e.g., board exam pass rates). Following the approach of Zhang et al., future work should contextualize model outputs relative to representative human baselines to provide a clearer interpretation of performance [13]. Comparative analyses between LLM-generated outputs and expert human opinions could also provide valuable insights into the strengths and limitations of these models in various clinical contexts. The rapid advancements in this field suggest that ongoing research will be crucial to understanding the evolving capabilities of LLMs in our field.

## Supporting information

S1 Table Summary of questions with an accuracy below 10% in all tested LLMs. Questions out of “Child and Adolescent Psychiatry for the Specialty Board Review”; 5^th^ Edition by Costello C, Schumacher L; Copyright © 2024. Reproduced by permission of Taylor & Francis Group.
(DOCX)

S2 Table Details about the four different models tested. All models are well established commercially available LLMs, accessed via the python interface of the providers openAI [1] and Google AI [2].
(DOCX)

S1 Fig Graphical assessment of normality (histograms). (A) represents the difference in accuracy between Gemini 1.5 Flash and Gemini 2.0 Flash. (B) represents the difference in accuracy between ChatGPT o1-mini and 4o. (C) represents the difference in accuracy between Gemini 2.0 Flash and ChatGPT o1-mini. (D) represents the difference in accuracy between Gemini 2.0 Flash and ChatGPT 4o.
(DOCX)

S2 Fig Graphical assessment of normality (boxplots). (A) represents the difference in accuracy between Gemini 1.5 Flash and Gemini 2.0 Flash. (B) represents the difference in accuracy between ChatGPT o1-mini and 4o. (C) represents the difference in accuracy between Gemini 2.0 Flash and ChatGPT o1-mini. (D) represents the difference in accuracy between Gemini 2.0 Flash and ChatGPT 4o.
(DOCX)

S1 Appendix Shapiro-Wilk test results. (DOCX)

S2 Appendix Wilcoxon signed-rank test results. (DOCX)
