# Supplementary material for: Performance of large language models ChatGPT and Gemini in child and adolescent psychiatry knowledge assessment
Source: PLoS One. 2025 Sep 19;20(9):e0332917. doi: 10.1371/journal.pone.0332917 (PMC12449005; doi:10.1371/journal.pone.0332917)
Supplement: S1 Table — Questions out of “Child and Adolescent Psychiatry for the Specialty Board Review”; 5th Edition by Costello C, Schumacher L; Copyright © 2024. Reproduced by permission of Taylor & Francis Group. (DOCX) [file pone.0332917.s001.docx]

**S1 Table: Summary of questions with an accuracy below 10% in all tested LLMs.** Questions out of “Child and Adolescent Psychiatry for the Specialty Board Review”; 5th Edition by Costello C, Schumacher L; Copyright © 2024. Reproduced by permission of Taylor & Francis Group.

| 1 | Which of the following neurotransmitters can impact coordinating the modulation of the entire neocortex and regulate theta rhythms on EEG? | Dopamine | Serotonin | Norepinephrine and epinephrine | Histamine | Acetylcholine |
| --- | --- | --- | --- | --- | --- | --- |
| 2 | At which of the following age ranges do children start to show an understanding of object permanence? | Age of 0–2 months | Age of 2–7 months | Age of 7–18 months | Age of 18–36 months | Age of 36–48 months |
| 3 | A meta-analysis by Cipriani et al. (2016) found the strongest association with increased risk of suicidality for children and adolescents given which of the following antidepressants? | Fluoxetine | Citalopram | Escitalopram | Sertraline | Venlafaxine |
| 4 | All of the following statements about the epidemiology of depressive disorders in children and adolescents are accurate except: | Point prevalence of depressive disorder is lower in prepubertal children than in adolescents | In adolescents, the rate of depressive disorders in females is three times more than in males | Pre-pubertal depressive disorder shares similar risk factors and course with conduct disorder | Pre-pubertal onset depressive disorder is more likely to lead to recurrent episodes in adulthood | Early onset of puberty is a risk factor for girls to develop depression |
| 5 | All of the following statements regarding the differential diagnoses and specific features of separation anxiety disorder and social anxiety disorder (social phobia) are correct except: | Increased sensitivity to carbon dioxide (CO2) exposure is found in children with separation anxiety disorder but not in children with social anxiety disorder (social phobia). | School refusal in children with social anxiety disorder is due to the fear of being negatively judged by others, not due to worries about being separated from attachment figures | Children with separation anxiety disorder are generally comfortable in social settings as long as they are accompanied by the attachment figures | Children with separation anxiety disorder may show temperamental characteristics of behavioral inhibition to the unfamiliar as young as 21 months | Children with social anxiety disorder (not with separation anxiety disorder) exhibit increased fear response when challenged in the laboratory with social stressors |
| 6 | Which of the following neurotransmitter systems has been shown to be an important genetic contributor for generalized anxiety disorder (GAD)? | Serotonin transporter genes | Serotonin receptor genes | Dopamine transporter genes | Noradrenergic receptor genes | None of the above |
| 7 | Which of the following types of child maltreatments may be most prevalent? | Emotional maltreatment (psychological maltreatment) | Physical abuse | Neglect | Sexual abuse | Sexual assault |
| 8 | In comparison of the prevalence rates of comorbid conditions between youth with borderline personality disorder and those without, which of the following conditions is significantly more prevalent in youth with borderline personality disorder? | ADHD | Conduct disorder | Major depressive disorder | Oppositional defiant disorder | Separation anxiety disorder |
| 9 | Which of the following SSRIs has the lowest incidence of sexual side effects? | Citalopram | Fluoxetine | Fluvoxamine | Paroxetine | Sertraline |
| 10 | All of the following antipsychotic-induced side effects are more prevalent in children and adolescents except: | Sedation | Akathisia | Withdrawal dyskinesia | Weight gain and metabolic abnormalities | Prolactin abnormalities |
| 11 | All of the following antidepressants have almost no sexual side effects except: | Bupropion | Duloxetine | Mirtazapine | Trazodone | Venlafaxine |
| 12 | Intoxication or withdrawal from which of the following substances appears to have the least effect on EEG? | Alcohol | Caffeine | LSD | Marijuana | Tobacco |
