# Supplementary material for: Performance of large language models ChatGPT and Gemini in child and adolescent psychiatry knowledge assessment
Source: PLoS One. 2025 Sep 19;20(9):e0332917. doi: 10.1371/journal.pone.0332917 (PMC12449005; doi:10.1371/journal.pone.0332917)
Supplement: S2 Table — All models are well established commercially available LLMs, accessed via the python interface of the providers openAI [1] and Google AI [2]. (DOCX) [file pone.0332917.s002.docx]

**S2 Table. Details about the four different models tested.** All models are well established commercially available LLMs, accessed via the python interface of the providers openAI [1] and Google AI [2].

| Model | | GPT-4o | o1-mini | Gemini 1.5 Flash | Gemini 2.0 Flash |
| --- | --- | --- | --- | --- | --- |
| Provider | openAI | | | Google AI | |
| Version | | gpt-4o-2024-08-06 | o1-mini-2024-09-12 | gemini-1.5-flash-002 | gemini-2.0-flash-001 |
| Price 1M Input / Output Tokens | | 2.50$ / 10.00$ | 1.10$ / 4.40$ | 0.075$ / 0.30 $ | 0.10$ / 0.40$ |
| Model card | | <https://openai.com/de-DE/index/gpt-4o-system-card/> | <http://openai.com/index/openai-o1-system-card/> | <https://storage.googleapis.com/deepmind-media/gemini/gemini_v1_5_report.pdf> | <https://storage.googleapis.com/model-cards/documents/gemini-2-flash.pdf> |

References:

[1] OpenAI API. OpenAI, 2025, <https://platform.openai.com/>

[2] Google AI API. Google, 2025, <https://ai.google/>
