# Supplementary material for: Performance of large language models ChatGPT and Gemini in child and adolescent psychiatry knowledge assessment
Source: PLoS One. 2025 Sep 19;20(9):e0332917. doi: 10.1371/journal.pone.0332917 (PMC12449005; doi:10.1371/journal.pone.0332917)

**S1 Figure. Graphical assessment of normality (histograms).** (A) represents the difference in accuracy between Gemini 1.5 Flash and Gemini 2.0 Flash. (B) represents the difference in accuracy between ChatGPT o1-mini and 4o. (C) represents the difference in accuracy between Gemini 2.0 Flash and ChatGPT o1-mini. (D) represents the difference in accuracy between Gemini 2.0 Flash and ChatGPT 4o.


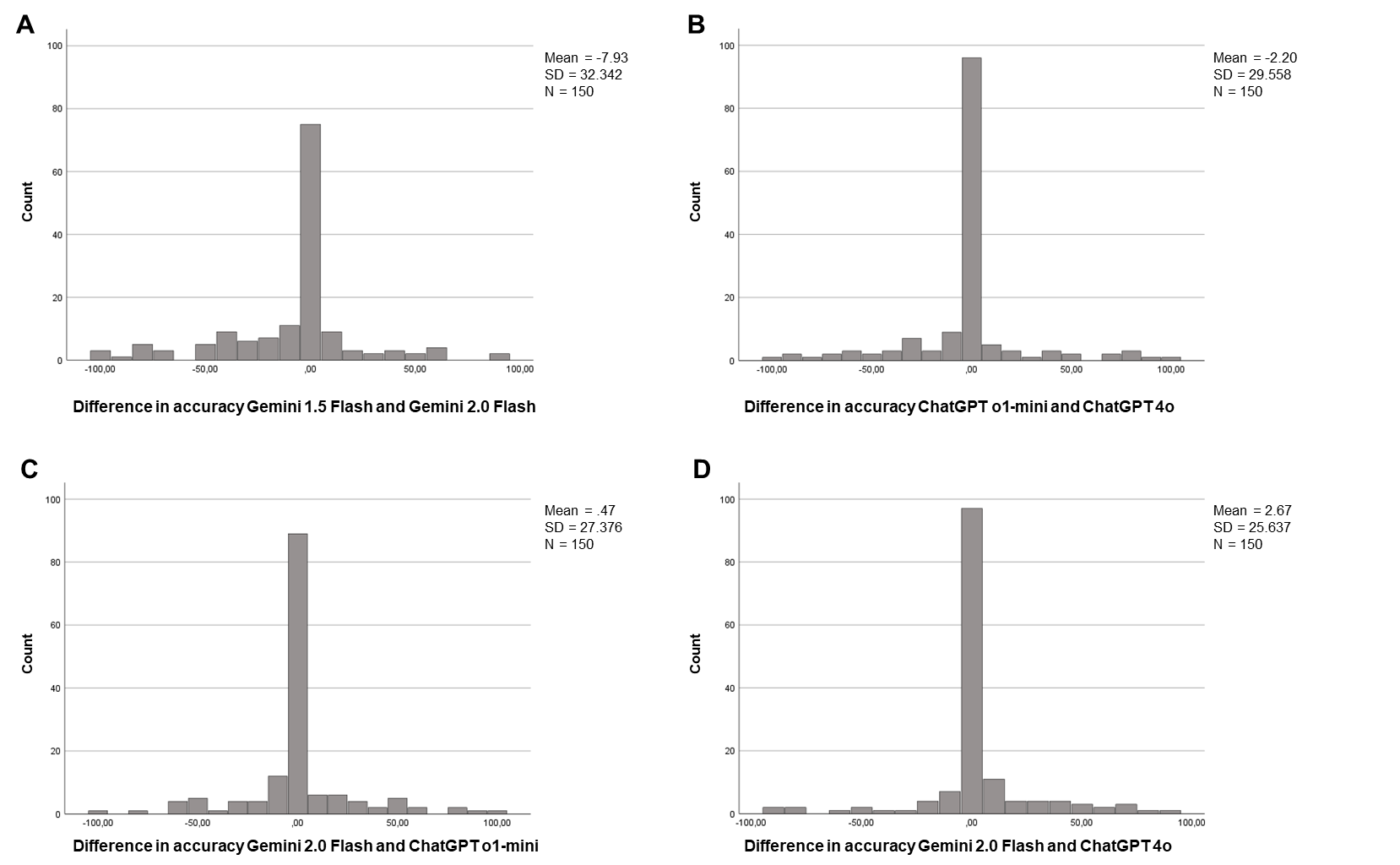

Supplement: S1 Fig — (A) represents the difference in accuracy between Gemini 1.5 Flash and Gemini 2.0 Flash. (B) represents the difference in accuracy between ChatGPT o1-mini and 4o. (C) represents the difference in accuracy between Gemini 2.0 Flash and ChatGPT o1-mini. (D) represents the difference in accuracy between Gemini 2.0 Flash and ChatGPT 4o. (DOCX) [file pone.0332917.s003.docx]
