# Supplementary material for: Performance of large language models ChatGPT and Gemini in child and adolescent psychiatry knowledge assessment
Source: PLoS One. 2025 Sep 19;20(9):e0332917. doi: 10.1371/journal.pone.0332917 (PMC12449005; doi:10.1371/journal.pone.0332917)
Supplement: S1 Appendix — (DOCX) [file pone.0332917.s005.docx]

**S1 Appendix: Shapiro-Wilk test results.**

Normality of the difference scores between variable Gemini 1.5 Flash and variable Gemini 2.0 Flash was assessed using the Shapiro–Wilk test, which revealed a significant deviation from normality (W = 0.857, p < 0.001). Also for the other comparisons, the Shapiro-Wilk test indicated that the difference scores deviated significantly from normality: ChatGPT o1-mini and ChatGPT 4o (W = 0.755, p < 0.001), ChatGPT o1-mini and Gemini 2.0 Flash (W = 0.797, p < 0.001) and ChatGPT 4o and Gemini 2.0 (W = 0.727, p < 0.001). Therefore, we also computed the corresponding non-parametric test to verify the robustness of the results.
