# Supplementary material for: Performance of large language models ChatGPT and Gemini in child and adolescent psychiatry knowledge assessment
Source: PLoS One. 2025 Sep 19;20(9):e0332917. doi: 10.1371/journal.pone.0332917 (PMC12449005; doi:10.1371/journal.pone.0332917)
Supplement: S2 Appendix — (DOCX) [file pone.0332917.s006.docx]

**S2 Appendix: Wilcoxon signed-rank test results.**

Comparing the accuracy of the models via Wilcoxon signed-rank test revealed no significant differences except for the comparison between Gemini 1.5 Flash and Gemini 2.0 Flash (Z = 2.99, p = .003, r = .24) in line with the results obtained from the t-tests.
